# Regular Endurance Exercise Promotes Fission, Mitophagy, and Oxidative Phosphorylation in Human Skeletal Muscle Independently of Age

**DOI:** 10.3389/fphys.2019.01088

**Published:** 2019-08-22

**Authors:** Estelle Balan, Céline Schwalm, Damien Naslain, Henri Nielens, Marc Francaux, Louise Deldicque

**Affiliations:** ^1^Institute of Neuroscience, Université catholique de Louvain, Louvain-la-Neuve, Belgium; ^2^Saint-Luc University Hospital, Université catholique de Louvain, Brussels, Belgium

**Keywords:** mitochondria, fusion, OXPHOS, mitogenesis, physical activity, endurance exercise

## Abstract

This study investigated whether regular endurance exercise maintains basal mitophagy and mitochondrial function during aging. Mitochondrial proteins and total mRNA were isolated from vastus lateralis biopsies (*n* = 33) of young sedentary (YS), old sedentary (OS), young active (YA), and old active (OA) men. Markers for mitophagy, fission, fusion, mitogenesis, and mitochondrial metabolism were assessed using qRT-PCR, Western blot, and immunofluorescence staining. Independently of age, fission protein Fis1 was higher in active vs. sedentary subjects (+80%; *P* < 0.05). Mitophagy protein PARKIN was more elevated in OA than in OS (+145%; *P* = 0.0026). mRNA expression of Beclin1 and Gabarap, involved in autophagosomes synthesis, were lower in OS compared to YS and OA (*P* < 0.05). Fusion and oxidative phosphorylation proteins were globally more elevated in the active groups (*P* < 0.05), while COx activity was only higher in OA than in OS (*P* = 0.032). Transcriptional regulation of mitogenesis did not vary with age or exercise. In conclusion, physically active lifestyle seems to participate in the maintenance of lifelong mitochondrial quality control by increasing fission and mitophagy.

## Introduction

Loss of muscle mass and strength is a well-known feature of aging in humans (Kim and Choi, 2013). Yet, direct and robust evidence is lacking to unveil the molecular mechanisms of aging in human, in particular in skeletal muscle function. In this perspective, much attention has been paid to mitochondria as skeletal muscle is highly dependent on mitochondrial oxidative phosphorylation to generate energy in the form of ATP (Egan and Zierath, 2013). ATP production is accompanied by the release of free radicals, which are deleterious for mitochondria when overwhelming antioxidant defenses, causing oxidative damage and impaired oxidative metabolism (Pinto and Moraes, 2015). Accumulation of mutant mitochondrial DNA (mtDNA) and free radicals during aging is both considered to negatively affect mitochondrial function, even though the correlation between those two processes is not clearly established (Pinto and Moraes, 2015). The maintenance of functional mitochondrial machinery is therefore probably decisive for skeletal muscle mass, quality, and function during aging (Seo et al., 2016).

Mitochondria are subjected to different quality control systems such as mitophagy, a selective autophagy degradation process, which specifically targets defective mitochondria (Ashrafi and Schwarz, 2013). Severely damaged/dysfunctional components of these organelles are first isolated from the healthy mitochondria pool by fission, trapped into an autophagosome, which then fuses with a lysosome endowed with hydrolases, enabling the disposal of mitochondria. An accumulation of damaged proteins specific to the mitochondrial compartment is thought to occur in skeletal muscle of old rodents and humans (Choksi et al., 2008; Wohlgemuth et al., 2010; Beltran Valls et al., 2015). This accumulation could underlie the failure of mitochondrial protein turnover, either through impaired mitochondrial biogenesis (mitogenesis) or mitochondrial degradation (mitophagy). Short-term assessment of mitochondrial protein expression using isotope labeling suggests that mitogenesis is reduced in older adults (Rooyackers et al., 1996). On the contrary, long-term isotope labeling and mitochondrial protein isolation indicates that lifelong mitochondrial protein synthesis is conserved in skeletal muscle of old mice, disputing the assumption that decreased mitogenesis is a feature of aging (Miller et al., 2012; Karunadharma et al., 2015). On the other hand, increased mtDNA alterations, abnormal reactive oxygen species (ROS) generation, and lessened oxidative capacity have been reported within mitochondria isolated from aged human skeletal muscle (Conley et al., 2000; Tonkonogi et al., 2003; Bua et al., 2006; Ghosh et al., 2011), evoking a decline in mitochondria quality control and function during aging. Yet, how these processes are affected during aging is far to be elucidated. Studies investigating the fate of mitochondria in aged skeletal muscle revealed numerous discrepancies, not only because of age heterogeneity between and within young and elderly participants, but also because analyses are mostly based on whole cellular extracts instead of mitochondrial fraction. As we previously evidenced (Schwalm et al., 2015, 2017), studying mitophagy in whole cellular extracts does not accurately reflect this process as mitophagy regulation relies on specific proteins transiting between cytosol and mitochondria.

Long-lasting exercise is recognized to improve global mitochondrial content and metabolism (Egan and Zierath, 2013; Hawley et al., 2014). In sedentary elderly, proteomics investigations revealed that exercise training increased electron transport chain proteins content and activity in mitochondria-enriched fraction (Menshikova et al., 2006; Ghosh et al., 2011). Since physical activity decreases during aging, the effect of this variable on mitochondrial integrity is difficult to isolate from aging *per se* when subjects are not matched for daily physical activity (Johannsen et al., 2008). Exercise is also currently emerging as a potential candidate to maintain mitochondria quality control. Indeed, acute endurance exercise has been shown to enhance fission and fusion markers in mitochondrial extracts of young active (YA) adults, as well as the expression of genes related to mitophagy (Cartoni et al., 2005; Schwalm et al., 2017). Moreover, endurance training has been suggested to promote fusion and fission in sedentary subjects independently of age (Konopka et al., 2014), although protein analyses were not specific to the mitochondrial compartment in the cited study. In summary, the understanding of the molecular mechanisms underlying the maintenance of mitochondrial integrity in human skeletal muscle with age is still in its infancy.

Here, we hypothesized that basal mitochondrial oxidative phosphorylation and quality control would be impaired with physical inactivity in human skeletal muscle during aging. More particularly, we hypothesized that mitochondrial oxidative phosphorylation and mitophagy in skeletal muscle would be reduced in sedentary old compared to young men, and that regular endurance exercise would prevent this decline.

## Materials and Methods

### Subject Characteristics

Thirty-four healthy men were recruited on voluntary basis (Table 1). Any subject with smoking history was excluded from the experiment. The protocol was approved by the local Ethical Committee of the Université catholique de Louvain and conducted in accordance with the Declaration of Helsinki. All participants provided their written consent after being fully informed about the experimental procedure. Subjects were divided into four groups: young sedentary (YS), old sedentary (OS), YA, and old active (OA) according to their age and self-reported weekly physical activity. The mean age of young subjects (YS + YA) was 22 ± 1 years and the mean age of old subjects (OS + OA) was 67 ± 1 years. None of the sedentary subjects was engaged in any weekly physical activity session for at least 5 years whereas physically active subjects were all experienced cyclists and reported ≥6 h training a week for at least 5 years. Before the beginning of the study, subjects reported to the laboratory for a medical examination aiming at excluding any underlying pathology. Afterward, height, weight, and body mass index (BMI) were determined. Subjects under medication related to blood pressure or cholesterol were excluded from the study. Participants were asked not to consume any dietary supplement and to refrain from strenuous activity during the 2 days preceding the experiment. The day before biopsy sampling, the diet of the subjects consisted in daily routine meals.

**TABLE 1:** Characteristics of the subjects.

	**Young**	**Old**	**Young**	**Old**
	**sedentary**	**sedentary**	**active**	**active**
	**(YS)**	**(OS)**	**(YA)**	**(OA)**
*N*	9	8	9	8
Age (y)	22 ± 1	67 ± 2^*⁣**,###^	22 ± 1	68 ± 1^*⁣**,###^
Height (cm)	182 ± 2	182 ± 2	182 ± 2	177 ± 3
Weight (kg)	76.4 ± 4.7^¶^	86.7 ± 3.8	76.8 ± 3.8^¶^	77.1 ± 2.8
BMI (kg m^–2^)	23.0 ± 1.1	26.4 ± 1.4^∗^	23.1 ± 1.0	24.6 ± 0.8
VO_2__peak_	48 ± 4	30 ± 2^*⁣**,###^	61 ± 3^∗∗^	44 ± 4^##,[$][$]^
(ml min^–1^ kg^–1^)				
W_max_ (W kg^–1^)	3.0 ± 0.2	1.9 ± 0.2^**,###^	4.3 ± 0.3^*⁣**^	3.3 ± 0.2^#,[$]^

*BMI, body mass index; VO_2__*peak*_, peak oxygen consumption; W_*max*_, maximal power output. Values are expressed as means ± SEM. ^∗^*P* < 0.05, ^∗∗^*P* < 0.01, ^∗∗∗^*P* < 0.001 vs. young sedentary group; ^#^*P* < 0.05, ^##^*P* < 0.01, ^###^*P* < 0.001 vs. young active group; ^$^*P* < 0.05 vs. old sedentary group; ^¶^0.01 < *P* < 0.05 vs. old sedentary group. ^$$^*P* < 0.01 vs. old sedentary group.*

### Cardiorespiratory Fitness Assessment

A maximal incremental exercise test was performed on a cycle ergometer (Cyclus III; RBM Electronics, Leipzig, Germany) for assessing cardiorespiratory fitness, as determined by peak oxygen consumption (VO_2__peak_) and maximal power output (W_max_). For YS, YA, and OA groups, the starting load was 70 W, incremented by 40 W every 3 min until exhaustion. For the OS group, the starting load was 30 W, incremented by 20 W every 3 min until exhaustion. Heart rate (Polar Team System 2; Polar Electro, Kempele, Finland) and respiratory exchanges (Ergocard Clinical, Medisoft, Sorinnes, Belgium) were continuously monitored.

### Skeletal Muscle Biopsy Sampling

A biopsy sample was taken in the fasted state from the mid portion of the *vastus lateralis* muscle (right leg) under local anesthesia (1 ml of Xylocaine 2%, AstraZeneca, Belgium) with a Bergström needle. About 80 mg of muscle was immediately frozen in liquid nitrogen for protein and RNA isolation, whereas about 20 mg was embedded in optimum cutting temperature (OCT) compound and immediately frozen in cooled isopentane to measure COx activity.

### Cytosolic and Mitochondrial Protein Fractionation

Approximately 20 mg of muscle was dedicated to cytosolic and mitochondrial protein fractionation according to Quadrilatero and Rush (2006) and as detailed in Schwalm et al. (2017). Cytosolic and mitochondrial protein amounts were determined using the DC kit for protein dosage (Bio-Rad Laboratories, Hercules, CA, United States). The purity of our extracts was verified based on the sole presence of glucose transport-4 (GLUT4/SCLC2A) and mitochondrial 20 kDa outer membrane protein (TOM20) protein in the cytosolic and mitochondrial fraction, respectively (Supplementary Figure 1A). GLUT4 was detected in the cytosolic fraction only whereas TOM20 was measured exclusively in the mitochondrial fraction, excluding any possible contamination between the two different fractions.

### SDS–PAGE and Immunoblotting

Approximately 20 μg of muscle proteins was mixed with Laemmli sample buffer and warmed for 5 min at 95°C. Proteins were separated by SDS–PAGE during 2 h at 40 mA, then transferred on polyvinylidene fluoride (PVDF) membranes for 2.5 h at 80 V. After blocking for 1 h in a Tris-buffered saline plus 0.1% Tween-20 (TBST) containing 5% non-fat dry milk, membranes were incubated at 4°C overnight with one of the following antibodies: Bcl-2/adenovirus E1B 19-kDa interacting protein 3-like (BNIP3) (ab10433), LAMP2b (ab118959), total oxidative phosphorylation (OXPHOS) cocktail (ab110413), parkin (ab77924) (Abcam, Cambridge, United Kingdom), autophagy protein 5 (Atg5) (#2630), citrate synthase (CS) (#14309), mitofusin-2 (#9482), TOM20 (#42406), optic atrophy 1 (OPA1) (#67589) (Cell Signaling Technology, Leiden, Netherlands), fission protein 1 (Fis1) (SAB2702049), microtubule-associated protein-1 light chain 3 (LC3b) (L7543) or p62/SQSTM1 (P0067) (Sigma–Aldrich, St. Louis, MO, United States), and GLUT4/SCLC2A (3G10A4) (Thermo Fisher Scientific, Waltham, MA, United States). Membranes were washed 3 × 10 min in TBST, then incubated for 1 h at room temperature with a secondary antibody conjugated to horseradish peroxidase. Three additional washes were done prior to chemiluminescence detection with the WesternBright Quantum HRP substrate (Advansta, Menlo Park, CA, United States). The bands were captured with the GeneSnap and quantified with the GeneTools software (G-Box, Syngene, Cambridge, United Kingdom). Atg5 and LAMP2b were quantified in the cytosolic fraction; LC3b and p62/SQSTM1 were both quantified in the cytosolic and mitochondrial fractions and all other protein expressions were quantified in the mitochondrial fraction. All protein expressions were reported to Coomassie blue density quantified on the membranes, which was equal between all experimental conditions. An internal standard was used to limit variations in protein expression when protein samples were separated on different gels.

### RNA Extraction and Quantitative Real-Time PCR

Approximately 30 mg of muscle biopsy was homogenized in 1 ml Trizol^®^ reagent (Invitrogen, Merelbeke, Belgium). RNA isolation was achieved according to the manufacturer’s instructions. RNA quality and quantity were assessed by Nanodrop^®^ spectrophotometry. Reverse transcription was performed from 1 μg RNA using the iScript^TM^ cDNA Synthesis Kit from Bio-Rad Laboratories, Hercules, CA, United States, following manufacturer’s instructions. The primer sequences are given in Table 2. PCR analyses were conducted using the following conditions: 2 min at 95°C, followed by 5 s at 95°C and 30 s at 60°C. All samples were run in triplicate with an internal standard on each plate to correct for interplate variability. Each reaction was processed in a 10-μl volume containing 4.8 μl SsoAdvanced Universal SYBR Green SuperMix (Bio-Rad Laboratories, Hercules, CA, United States), 0.1 μl of each primer (100 nM final), and 5 μl cDNA at the appropriate dilution. Melting curves were systematically performed for quality control. According to Vandesompele et al. (2002) method, relative mRNA levels were normalized to beta-2-microglobulin (*B2M*), ribosomal protein L19 (*RPL-19*), and ribosomal protein L4 (*RPL-4*), whose geometrical mean expression was unaffected by age or physical activity.

**TABLE 2:** Primer sequences.

	**Forward**	**Reverse**
Beclin1	5′-CACATCTGGCACAGTGGACA-3′	5′-CGGCAGCTCCTTAGATTTGT-3′
Gabarap	5′-GTGCCCTCTGACCTTACTGTTG-3′	5′-CATTTCCCATAGACACTCTCATC-3′
NRF1	5′-GCAAGCTATTGTCCTCTGTATC-3′	5′-GTACTTACGCACCACATTCTC-3′
NRF2	5′-AAACTTCTGTTG CTCAGGTAG-3′	5′-TAAGACACTGTAACTCAGGAATG-3′
p62	5′-CCTCTGGGCATTGAAGTTG-3′	5′-TATCCGACTCCATCTGTTCCTC-3′
PGC1α	5′-CACTTACAAGCCAAACCAACAACT-3′	5′-GTGCGAACCACTCTTAGATAC-3′
Tfam	5′-CTCAGAACCCAGATGCAAA-3′	5′-GCCACTCCGCCCTATAA-3′

*Beclin1; Gabarap, gamma-aminobutyric acid receptor-associated protein; NRF1, nuclear respiratory factor 1; NRF2, nuclear factor erythroid-derived-2-like 2; p62, ubiquitin-binding protein p62; PGC1α, peroxisome proliferator-activated receptor gamma coactivator 1-alpha; and Tfam, mitochondrial transcription factor A.*

### DNA Extraction and Quantitative PCR

Total DNA was isolated from biopsy samples using PureLink^®^ Genomic DNA Kit (Life Technologies, Carlsbad, CA, United States) according to manufacturer’s instructions. Assessment of DNA quality and quantity, as well as PCR analyses was conducted similarly as described in the section above for RNA and cDNA, respectively. The amount of mtDNA and nuclear DNA was quantified using primers *MT-COI* forward sequence 5′-CCCTGCCATAACCCAATAC-3′ and reverse sequence 5′-CTGGGAGAGATAGGAGAAGTAG-3′ and primers *RPL19* forward sequence 5′-CGCTGTGGCAAGAAGA AGGTC-3′ and reverse sequence 5′-GGAATGGACCGTCAC AGGC-3′.

### COx Activity

Cryosections (6 μm) were air-dried and incubated in phosphate buffer 0.05 M pH 7.3 containing 20 mg of diaminobenzidine, 140 mg of cytochrome C, 3 g of saccharose, and 4 ml of catalase (Sigma–Aldrich, St. Louis, MO, United States). Slides were then rinsed in distilled water, dehydrated in three alcohol baths (95°, 100°, and 100°), fixed in Xylene before being mounted with Eukitt^®^ Quick-hardening medium (Sigma–Aldrich, St. Louis, MO, United States). Measurements of COx intensity were performed by converting the image to gray scale to determine optical density. Images were acquired in the same exposure conditions using Zeiss Primo Vert microscope with a 10× objective. The mean relative optical density per pixel was determined by subtracting the optical density of the background using ImageJ software. For each subject, a single value was obtained by averaging measurements from approximately 100 muscle fibers, which were chosen at random.

### Statistics

All values are expressed as the means ± the standard error of the mean (SEM). Statistical analyses were performed using GraphPad Prism 7.0. Data were analyzed by two-way ANOVA to test the main effect of age and physical activity, as well as the interaction between these two factors. When a significant main effect was found, Fisher *post hoc* tests were performed to test potential differences between sedentary and active subjects within the same age group and differences between young and elderly subjects with the same level of physical activity. Statistical significance was set at *P* < 0.05.

## Results

### Subjects Characteristics

Subjects’ characteristics are described in Table 1. Height and weight were not different between groups. BMI was higher with age (main effect of age, *P* = 0.039). *Post hoc* analysis showed that BMI was higher in OS compared to YS (*P* = 0.039). VO_2__peak_ and W_max_ were lower with age and higher with physical activity (all main effects, *P* < 0.001). VO_2__peak_ was about 25% higher in YA compared to YS (*P* = 0.035) and about 50% higher in OA compared to OS (*P* = 0.029). OA had similar VO_2__peak_ values as YS (∼45 ml min^–1^ kg^–1^). Similarly, W_max_ was about 40% higher in YA compared to YS (*P* < 0.001) and about 75% higher in OA compared to OS (*P* < 0.001). OA had similar W_max_ values as YS (∼3 W kg^–1^).

### Regular Physical Activity Enhances Mitochondrial Markers of Fission, Mitophagy, and the Presence of Autophagosomes

**FIGURE 1:**
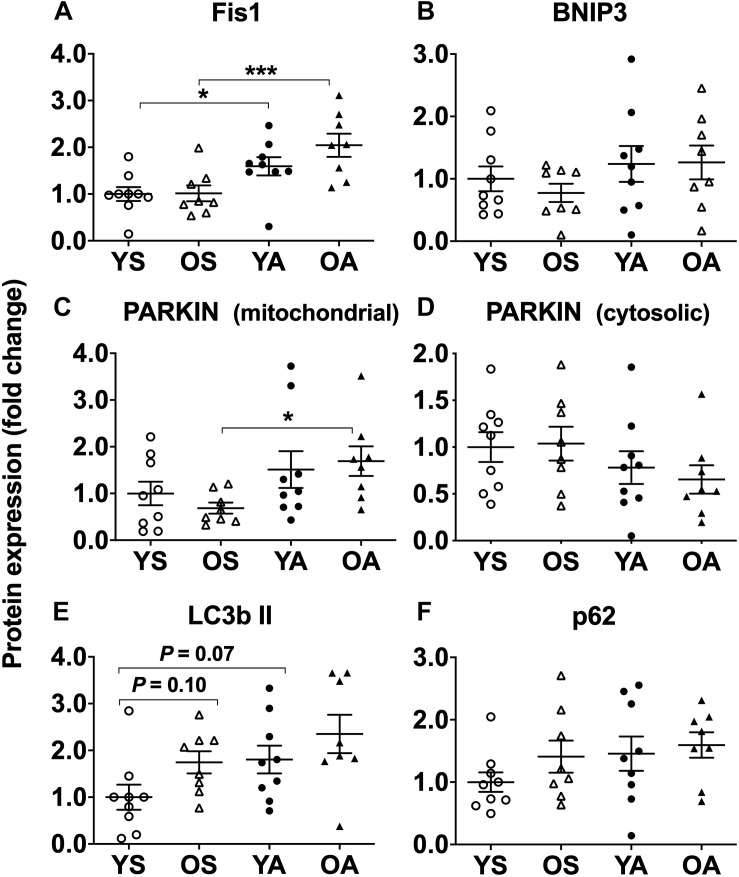
Regulation of fission and mitophagy protein markers by age and physical activity in human skeletal muscle. Mitochondrial protein expression of **(A)** Fis1, **(B)** BNIP3, **(C)** PARKIN, cytosolic protein expression of **(D)** PARKIN, mitochondrial protein expression of **(E)** LC3b II, and **(F)** p62/SQSTM1 in young and old men in response to sedentary vs. active lifestyle. YS, young sedentary; OS, old sedentary; YA, young active; OA, old active. Values are expressed as means ± SEM. *^∗^P* < 0.05, *^∗∗∗^P* < 0.001.

Independently of age, the protein expression of the fission marker Fis1 was higher in the active groups in comparison to the respective sedentary ones (main effect of physical activity, *P* < 0.001; *post hoc* YA vs. YS, *P* = 0.032; OA vs. OS, *P* < 0.001; Figure 1A and Supplementary Figure 1B). BNIP3, a mitochondrial receptor for LC3b (Rikka et al., 2011), was not affected by age or physical activity (Figure 1B and Supplementary Figure 1B). The presence of PARKIN in the mitochondrial fraction was more elevated in the active than in the sedentary subjects (main effect of physical activity, *P* = 0.026; *post hoc*, OA vs. OS, *P* = 0.026; Figure 1C and Supplementary Figure 1B), while the cytosolic expression of PARKIN tended to be lower (main effect of physical activity, *P* = 0.082; Figure 1D and Supplementary Figure 1B). The protein expression of LC3b II in the mitochondrial fraction was higher with age and physical activity (main effect of physical activity and age, *P* = 0.030 and *P* = 0.045, respectively). *Post hoc* analysis showed that there was a trend to higher LC3b II levels in YA vs. YS (*P* = 0.067) and in OS vs. YS (*P* = 0.098; Figure 1E and Supplementary Figure 1B). Mitochondrial LC3b II/I ratio, commonly assessed as an indicator of autophagosome synthesis, was not displayed here because the presence of LC3b I was almost undetectable in the mitochondrial fraction (data not shown). Mitochondrial p62/SQSTM1 protein expression, which targets mitochondrial protein aggregates and tethers LC3 to enable their elimination (Pankiv et al., 2007; Geisler et al., 2010), was similar among the groups (Figure 1F and Supplementary Figure 1B).

### Attenuated Transcriptional Regulation of Genes Implicated in Autophagosome Synthesis During Aging Is Reversed by Physical Activity

**FIGURE 2:**
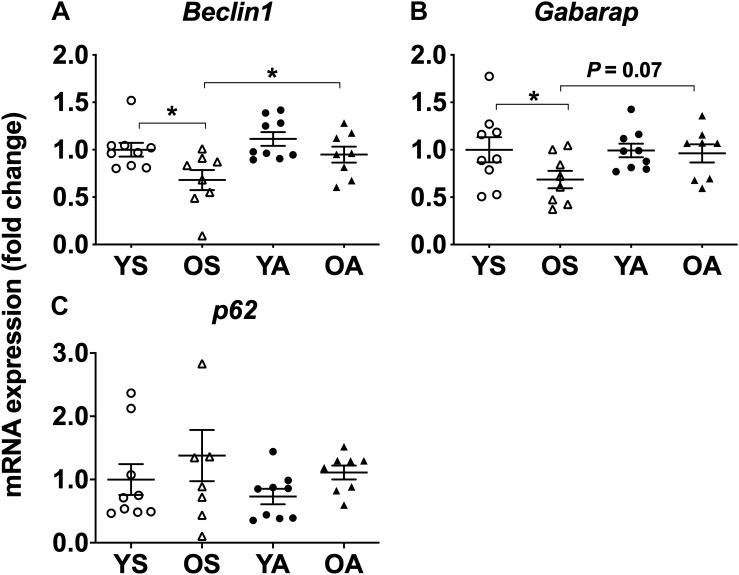
Regulation of mitophagy transcriptional markers by age and physical activity in human skeletal muscle. mRNA levels of **(A)** *Beclin1*, **(B)** *Gabarap*, and **(C)** *p62* in young and old men in response to sedentary vs. active lifestyle. YS, young sedentary; OS, old sedentary; YA, young active; OA, old active. Values are expressed as means ± SEM. *^∗^P* < 0.05.

Overall, mRNA expression of Beclin1 and Gabarap, coding for proteins linked to fusion between mature autophagosomes and lysosomes (Bento et al., 2016), was lower with age (main effect of age, *P* = 0.007 for Beclin1 and *P* = 0.023 for Gabarap) and higher with physical activity (main effect of physical activity, *P* = 0.030 for Beclin1 and *P* = 0.042 for Gabarap, Figures 2A,B). *Post hoc* analysis revealed that Beclin1 and Gabarap mRNA were lower in OS than in YS (*P* = 0.011 and *P* = 0.037, respectively). Moreover, OS exhibited lower Beclin1 expression in comparison with OA (*post hoc*, *P* = 0.035). *Post hoc* analysis also highlighted a trend to a lower Gabarap expression when OS were compared to OA (*P* = 0.072). p62 mRNA levels did not vary with age or physical activity (Figure 2C).

### Markers of Autophagy in the Cytosolic Fraction Are Barely Influenced by Aging and Physical Activity

The expression of autophagy-related proteins measured in the cytosolic fraction was not modified by age or exercise, except LC3b II, which was lower in the active groups (main effect of physical activity, *P* = 0.031) (Supplementary Figures 1C, 2A–F). According to *post hoc* analysis, this effect was mainly due to a two times lower expression of LC3b II in YA compared to YS (*P* = 0.033, Supplementary Figures 1C, 2C), which did not result in different LC3b II/I ratios (Supplementary Figure 2D). Globally, LAMP2b protein expression was lower in the active groups (main effect of physical activity, *P* = 0.045) (Supplementary Figures 1C, 2F). *Post hoc* analysis only revealed a trend to LAMP2b downregulation in OA compared to OS (*P* = 0.074).

### Regular Physical Activity Increases Mitochondrial Fusion in Old Men

**FIGURE 3:**
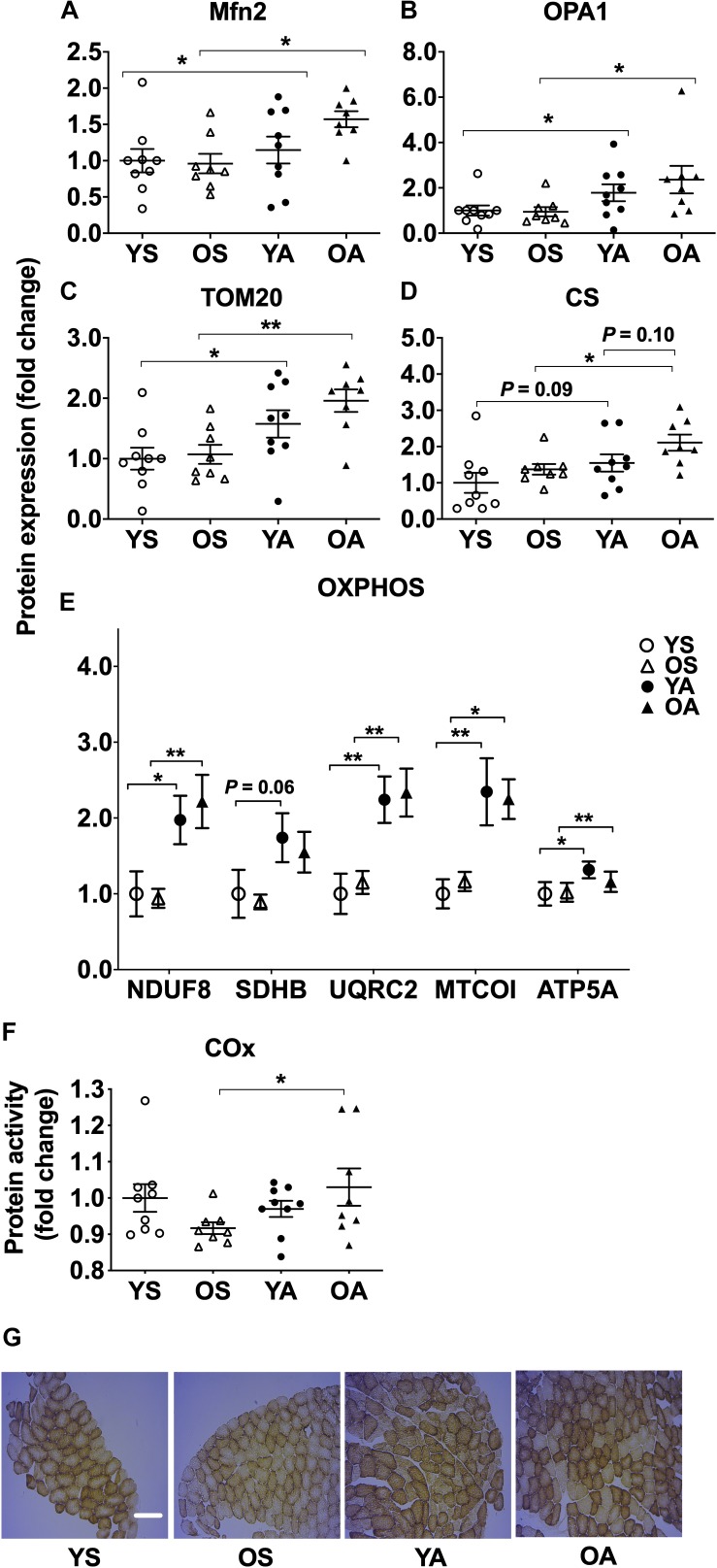
Regulation of fusion, import, and oxidative phosphorylation protein markers by age and physical activity in human skeletal muscle. Mitochondrial protein expression of **(A)** Mfn2, **(B)** OPA1, **(C)** TOM20, **(D)** CS, **(E)** OXPHOS, and **(F)** protein activity of COx in young and old men in response to sedentary vs. active lifestyle. YS, young sedentary; OS, old sedentary; YA, young active; OA, old active. Values are expressed as means ± SEM. *^∗^P* < 0.05, ^∗∗^*P* < 0.01. **(G)** Representative muscle cross sections stained for COx protein activity according to age and physical activity. YS, young sedentary; OS, old sedentary; YA, young active; OA, old active. 100 fibers were analyzed per subject. Scale bar is 120 μM.

The protein expression of markers of mitochondrial fusion Mfn2 and OPA1 was higher in the mitochondrial fraction of the active subjects (main effect of physical activity, *P* = 0.020 and *P* = 0.007, respectively; *post hoc*, OA vs. OS: Mfn2, *P* = 0.013; OPA1, *P* = 0.016; YA vs. YS: Mfn2, *P* = 0.013; OPA1, *P* = 0.017) (Figures 3A,B and Supplementary Figure 1D).

### Regular Physical Activity Upregulates Mitochondrial Importer and Oxidative Phosphorylation Proteins Independently of Age

TOM20, involved in the import of proteins through the outer mitochondrial membrane (Harbauer et al., 2014), was more expressed in the mitochondrial fraction of the active groups (main effect of physical activity, *P* < 0.001; *post hoc*, OA vs. OS, *P* = 0.003; YA vs. YS, *P* = 0.037; Figure 3C and Supplementary Figure 1D). For CS protein expression, a main effect of physical activity (*P* = 0.009) and a tendency for age (*P* = 0.052) were detected (Figure 3D and Supplementary Figure 1D). CS expression was more elevated in OA than in OS (*P* = 0.037) and tended to be higher in YA vs. YS (*P* = 0.093) and in OA vs. YA (*P* = 0.097). Independently of age, a higher mitochondrial expression of oxidative phosphorylation complexes I (subunit NDUF8), III (subunit UQRC2), IV (subunit MTCOI), and V (subunit ATP5A) was found in the physically active groups (main effect of physical activity, *P* < 0.001), reaching a twofold higher expression compared to the sedentary groups (*post hoc*, NDUF8: YA vs. YS, *P* = 0.021; OA vs. OS, *P* = 0.005; UQRC2: YA vs. YS, *P* = 0.002; OA vs. OS, *P* = 0.005; MTCOI: YA vs. YS, *P* = 0.002; OA vs. OS, *P* = 0.016; ATP5A: YA vs. YS, *P* = 0.037; OA vs. OS, *P* = 0.003; Figure 3E and Supplementary Figure 1D). *Post hoc* analysis revealed that expression of oxidative phosphorylation complex II (subunit SDHB) tended to be higher in YA compared to YS (*P* = 0.059). COx activity was not different between YS, YA, and OA groups but was lower in OS compared to their active counterparts (*post hoc*, *P* = 0.032, Figures 3F,G).

### Regular Physical Activity Stimulates Mitochondria Copy Number but Canonical Genes Coding for Mitogenesis Proteins Are Only Slightly Modified by Age or Exercise Training

**FIGURE 4:**
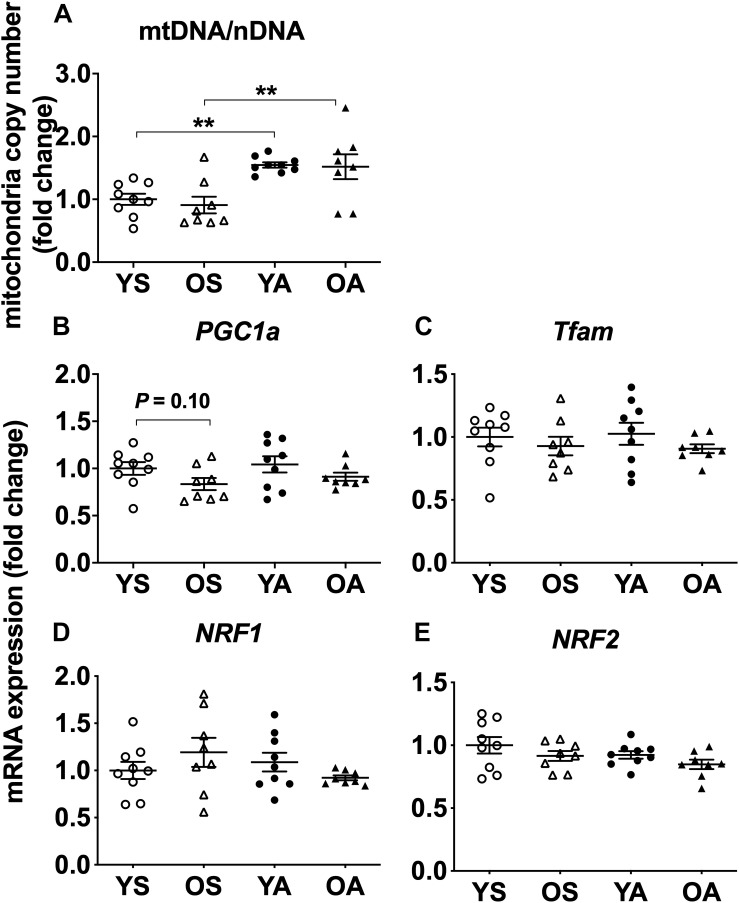
Regulation of mitogenesis transcriptional markers by age and physical activity in human skeletal muscle. Mitochondria copy number **(A)**, mRNA levels of **(B)** PGC1α, **(C)** Tfam, **(D)** NRF1, and **(E)** NRF2 in young and old men in response to sedentary vs. active lifestyle. YS, young sedentary; OS, old sedentary; YA, young active; OA, old active. Values are expressed as means ± SEM. *^∗∗^P* < 0.01.

Independently of age, the number of mitochondria copies assessed by the mtDNA/nDNA ratio was augmented by lifelong physical activity (main effect of physical activity, *P* < 0.001, Figure 4A). YA had a 1.5-fold higher mtDNA/nDNA ratio compared to YS (*post hoc*, *P* = 0.003). Similarly, mtDNA/nDNA was higher in OA than in OS (*post hoc*, *P* = 0.002). Globally, the mRNA expression of the genes related to mitogenesis was not different between age-matched or exercise-matched groups, excepted for PGC1α, which was less expressed in the older groups (main effect of age, *P* = 0.038, Figure 4B). According to *post hoc* analysis, PGC1α mRNA levels tended to be lower in OS in comparison to YS (*P* = 0.097). The mRNA levels of Tfam (Figure 4C), NRF1 (Figure 4D), and NRF2 (Figure 4E) were not different between the groups.

## Discussion

To our knowledge, this study is the first to investigate the effect of regular endurance exercise on basal markers of mitophagy, mitogenesis, and oxidative phosphorylation during aging, in the mitochondrial fraction of human muscle biopsies. The inclusion of subjects matched for age and physical activity allows to distinguish the effect of age and regular physical activity on the outcomes. Our findings revealed that mitochondrial protein markers related to these processes are not affected by chronological age *per se* but are largely improved by regular endurance exercise. They highlight the importance of maintaining a physically active lifestyle during aging for preserving mitochondria quality control.

### Regular Physical Activity Influences Mitochondrial Quality Control Markers

As we recently observed that acute endurance exercise promoted fission in mitochondrial-enriched muscle fraction of young athletes, we assessed the effects of long-term exercise on mitochondrial quality control in young adults compared to their old counterparts, including sedentary groups matched for age as controls (Schwalm et al., 2017). In agreement with a previous experiment performed in whole muscle extracts of young and OS subjects, we confirmed in the mitochondrial fraction that regular endurance exercise increases mitochondrial fission regardless of chronological age, since Fis1 was higher in both physically active groups (Konopka et al., 2014). However, the level of Fis1 was not different between our two sedentary groups, indicating that regulation of fission is probably not affected in subjects of 65–70 years old. As aging favors the accumulation of defective mitochondria (Conley et al., 2000; Tonkonogi et al., 2003; Bua et al., 2006; Ghosh et al., 2011), this observation suggests that muscle cell is not spontaneously able to upregulate fission to fight against the accumulation of dysfunctional mitochondria, which has been proposed to be due to mtDNA changes, impaired mitochondrial protein synthesis, and/or decreased activity of individual mitochondrial enzymes and enzymes complexes, among others (Short and Nair, 2001). In summary, our results point to a likely contribution of regular endurance exercise to mitochondria quality control, of particular interest in older subjects presenting dysfunctional mitochondria.

Fission being the preliminary step of mitophagy, we subsequently quantified downstream markers involved in specific recognition and removal of undesirable mitochondria. The lower amount of PARKIN and Fis1 in the mitochondrial fraction of OS compared to physically active subjects points toward an altered mitochondrial quality control related to physical inactivity during aging. These results are in agreement with a higher phospho-PARKIN^Ser65^ recently reported in whole muscle extracts of trained subjects (Tarpey et al., 2017). Here, based on mitochondrial fractionation, we provide additional evidence that regular exercise training promotes the translocation of PARKIN from the cytosol to the mitochondria in resting conditions.

LC3b II depicts autophagosomes abundance. It interacts with BNIP3 and p62/SQSTM1 to help the engulfment of mitochondria into autophagosomes (Birgisdottir et al., 2013). We previously reported an increased expression in *Bnip3*, *LC3b*, and *p62* mRNA after acute exercise in young people (Schwalm et al., 2017). Consequently, we expected that long-term physical activity would increase the expression of proteins coded by these genes (Schwalm et al., 2015, 2017). Neither BNIP3, nor p62/SQSTM1 content was modified in young and old healthy men. Nonetheless, recruitment of autophagosomes toward dysfunctional mitochondrial proteins has been proposed to be fostered by phosphorylation of BNIP3 and p62/SQSTM1 (Matsumoto et al., 2011; Zhu et al., 2013). For that reason, it cannot be excluded that regular exercise and/or aging could influence the phosphorylation state of these markers despite invariant total proteins levels. A doubled amount of mitochondrial LC3b II was observed in trained subjects in comparison to YS, while LC3b I, the immature form of LC3b, was almost undetectable in the mitochondrial fraction. These results fit with the chronological steps of mitophagy, since the generation of mature autophagosomes, reflected by LC3b II levels, is required to bind mitochondria before their degradation. Interestingly, a tendency to an elevated mitochondrial LC3b II content coincided with a reduced cytosolic level of the latter in YA, suggesting that autophagosomes are preferentially directed toward mitochondria in the young physically active group. Moreover, invariant cytosolic markers of autophagy in the active subjects compared to the sedentary ones were compatible with a selective degradation of mitochondria by regular exercise training. Altogether, those findings provide objective arguments in favor of an upregulation of mitophagy markers with exercise training.

To gain further insight into the regulation of mitochondria quality control according to age and physical activity, we measured the mRNA expression of Beclin1, Gabarap, and p62. All of them are regulated by the transcription factor EB (TFEB), which drives the expression of both autophagosomes and lysosomes synthesis-related genes upon its nuclear translocation in response to environmental cues, such as nutrient availability and cellular stress (Settembre et al., 2011). Lower Beclin1 and Gabarap expression, identified in OS only, may result from TFEB retention in the cytosol. Moreover, those changes would evoke an impaired transcriptional regulation of autophagosome formation and maturation, instigated by aging coupled to physical inactivity. Conversely, expression of Beclin1 and Gabarap was restored with active lifestyle during aging, suggesting that regular physical activity might have a protective effect on mitochondrial health.

### Regular Endurance Exercise Enhances Mitochondrial Dynamics Protein Content

To our knowledge, the effect of lifelong regular physical activity on fusion markers was not documented in mitochondria-enriched fraction of human skeletal muscle. Lower *Mfn2* found in sedentary seniors as well as enhanced *Mfn2* mRNA noted 24 h after acute endurance exercise in young subjects suggested that impaired fusion during aging may be improved with long-term exercise (Cartoni et al., 2005; Tezze et al., 2017). In our experiment, higher level of fusion proteins in physically active subjects fitted with several studies which reported augmented fusion with long-term exercise in whole muscle extracts of young and old adults (Konopka et al., 2014; Tezze et al., 2017; Arribat et al., 2018). Here we bring the first observation that regular endurance exercise upregulates fusion proteins expression, specifically in the mitochondrial fraction in human. Similar expression of fusion proteins between young and old subjects revealed that this process is independent of age, at least in the present conditions. Whereas fusion pulls mitochondria together into an interconnected network to preserve slightly faulty mitochondria from mitophagy, fission splits the mitochondrial pool to segregate and get rid of severely damaged organelles. Considering that the amounts of fission and fusion proteins were augmented in both young and OA subjects, fusion and fission likely acted as complementary processes in the adaptations to regular exercise training. In brief, our results suggest that regular exercise stimulates mitochondria dynamics protein expression, where both fusion and fission are needed to insure the health of the mitochondrial pool.

### Regular Endurance Exercise Promotes OXPHOS

Because they are encoded in the nucleus and translated in the cytosol, most of the mitochondrial proteins require entering mitochondria through an import system to which TOM20 contributes (Harbauer et al., 2014). Here, increased presence of proteins participating in mitochondrial dynamics and function in the mitochondrial fraction of active subjects, especially for aged ones, was likely facilitated by enhanced expression of TOM20. These findings imply that import of mitochondrial proteins would play a preeminent role in mitochondrial integrity and function during life.

Beyond its role in mitochondrial dynamics, fusion was believed to foster mitochondria metabolism through synthesis of coenzyme Q without changes in total OXPHOS (Mourier et al., 2015). Still, accrued expression of CS and OXPHOS proteins with physical activity during aging shows that regular exercise challenges mitochondrial oxidative phosphorylation at long term. Moreover, as OPA1 was reported to be responsible for mitochondrial cristae shape and length remodeling, increased OPA1 expression would be consistent with enhancement of mitochondrial oxidative phosphorylation markers (Tezze et al., 2017). In this view, augmented fusion proteins would go in hand with accrued presence of oxidative phosphorylation proteins and COx activity measured in old physically active subjects.

A recent study performed in murine skeletal muscle reported that nuclear translocation of TFEB in response to training coincided with an increased expression and activity of CS, COx, and OXPHOS (Mansueto et al., 2017), supporting that TFEB may mediate training-induced changes in oxidative phosphorylation. In human skeletal muscle, such observations have not been confirmed yet. Here, we found that long-term endurance exercise promoted CS and OXPHOS expression whatever the age, as well as COx activity in old men.

Markers of mitochondrial oxidative phosphorylation were not different between young and OA subjects. However, our senior active subjects exhibited a lower VO_2__peak_ compared to YA men. VO_2__peak_ is known to decline during aging, due to lower stroke volume and arterial-venous oxygen concentration (Strait and Lakatta, 2012). Therefore, increasing oxidative phosphorylation markers can be viewed as a mean to compensate for lessened cardiorespiratory flexibility during aging.

Regular endurance exercise is known to increase mitochondrial content in skeletal muscle. In our study, the basal expression of the main genes involved in mitogenesis was modified neither by aging nor by physical activity. This is in line with previous results showing no difference in PGC-1α mRNA between untrained and trained subjects in a resting state (Pilegaard et al., 2003). The genes implicated in mitochondrial biogenesis are transcribed during and within the few hours after exercise. In our experiment, the muscle biopsy samples were taken several days after the last exercise session. Thus, those results are compatible with increased mitochondrial copy number observed in the active subjects, which is commonly used to estimate mitogenesis and mitochondrial health (Medeiros, 2008). Nonetheless, they give arguments in favor of preserved mitochondria integrity with regular endurance exercise. Moreover, similar mtDNA/nDNA measured between young and old groups are questioning the assumption according to which mtDNA integrity would be altered during aging (Bua et al., 2006), at least in healthy men.

### Limitations

We have to acknowledge that the results obtained in the present study are based exclusively on markers for autophagy and mitophagy, which is an inherent limitation from human skeletal muscle biopsies. The measurement of autophagy flux is not possible, which precludes any direct assessment of autophagy activity. In addition, no distinction between fiber types was made. Whether autophagy and mitophagy are differently regulated in slow or fast fibers with aging remains therefore unanswered.

## Conclusion

In conclusion, the present study, based on mitochondrial extracts, demonstrates that expression of mitochondrial quality control and function markers does not depend on chronological age, but is improved by regular physical activity. These findings bring new pieces of evidence that mitochondrial fission and mitophagy are higher in skeletal muscle of physically active humans.

## Data Availability

All datasets for this study are included in the manuscript and the Supplementary Files.

## Ethics Statement

This study was carried out in accordance with the recommendations of the “Comité D’éthique Hospitalo-Facultaire (UCLouvain)” with written informed consent from all subjects. All subjects gave written informed consent in accordance with the Declaration of Helsinki. The protocol was approved by the “Comité D’éthique Hospitalo-Facultaire (UCLouvain).”

## Author Contributions

EB, CS, DN, and HN acquired the data. EB, CS, and DN analyzed the data. EB, CS, MF, and LD interpreted the results. MF and LD designed the study. CS and LD wrote the first draft of the manuscript. All authors approved the final version of the manuscript.

## Conflict of Interest Statement

The authors declare that the research was conducted in the absence of any commercial or financial relationships that could be construed as a potential conflict of interest.

## Supplementary Material

FIGURE S1 Illustrations of western blot. **(A)** Cytosolic and mitochondrial protein fractionation. GLUT-4/SCLCA and TOM20 verified respectively the purity of cytosolic and mitochondrial fraction. **(B)** Cytosolic expression of PARKIN and mitochondrial expression of FIS1, BNIP3, PARKIN, LC3B II, and p62 reported to Coomassie. **(C)** Cytosolic expression of Atg5, LC3b I, LC3b II, p62, and Lamp2b reported to Coomassie. **(D)** Mitochondrial expression of Mfn2, OPA1, TOM20, and CS reported to Coomassie. Mitochondrial expression of oxidative phosphorylation complexes I (subunit NDUF8), II (subunit SDHB), III (subunit UQRC2), IV (subunit MTCOI), and V (subunit ATP5A).

FIGURE S2 Markers of autophagy in the cytosolic fraction are barely influenced by aging and physical activity. Cytosolic expression of **(A)** Atg5, **(B)** LC3b I, **(C)** LC3B II, **(D)** LC3B II/I, **(E)** p62/SQSTM1, and **(F)** LAMP2b. Values are expressed as means ± SEM. ^∗^*P* < 0.05.
