# Supplementary figures and images for: Regular Endurance Exercise Promotes Fission, Mitophagy, and Oxidative Phosphorylation in Human Skeletal Muscle Independently of Age
Source: Front Physiol. 2019 Aug 22;10:1088. doi: 10.3389/fphys.2019.01088 (PMC6713923; doi:10.3389/fphys.2019.01088)

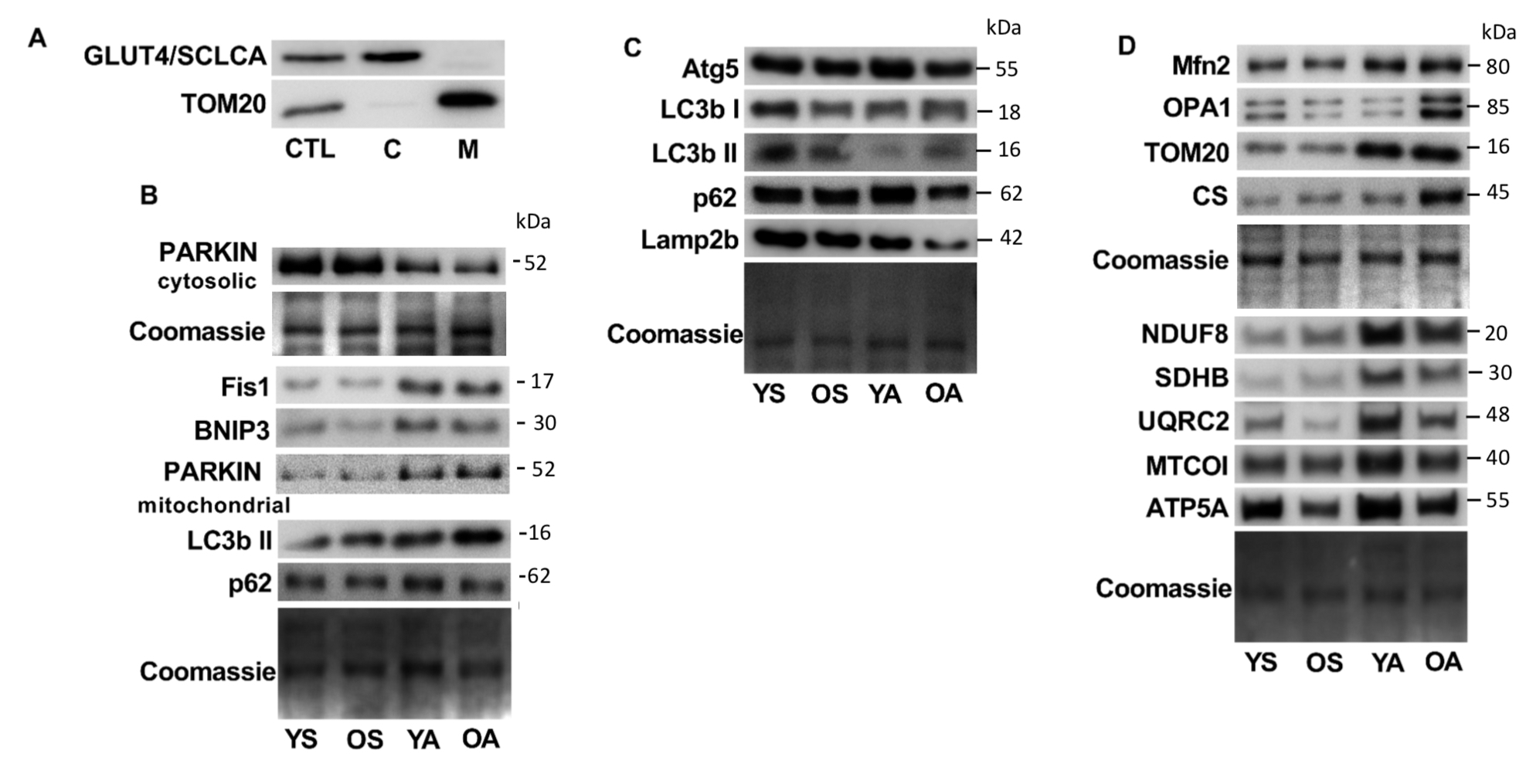

Supplement: FIGURE S1 — Illustrations of western blot. (A) Cytosolic and mitochondrial protein fractionation. GLUT-4/SCLCA and TOM20 verified respectively the purity of cytosolic and mitochondrial fraction. (B) Cytosolic expression of PARKIN and mitochondrial expression of FIS1, BNIP3, PARKIN, LC3B II, and p62 reported to Coomassie. (C) Cytosolic expression of Atg5, LC3b I, LC3b II, p62, and Lamp2b reported to Coomassie. (D) Mitochondrial expression of Mfn2, OPA1, TOM20, and CS reported to Coomassie. Mitochondrial expression of oxidative phosphorylation complexes I (subunit NDUF8), II (subunit SDHB), III (subunit UQRC2), IV (subunit MTCOI), and V (subunit ATP5A). [file Image_1.JPEG]

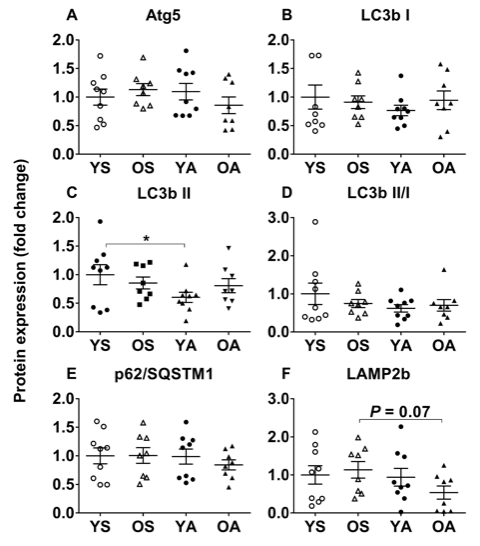

Supplement: FIGURE S2 — Markers of autophagy in the cytosolic fraction are barely influenced by aging and physical activity. Cytosolic expression of (A) Atg5, (B) LC3b I, (C) LC3B II, (D) LC3B II/I, (E) p62/SQSTM1, and (F) LAMP2b. Values are expressed as means ± SEM. ∗P < 0.05. [file Image_2.TIFF]
